# Feasibility study on recruitment in general practice for a low back pain online information study (part of the ADVIN Back Trial)

**DOI:** 10.1186/s13104-020-4894-8

**Published:** 2020-01-10

**Authors:** Allan Riis, Michael Skovdal Rathleff, Jan Hartvigsen, Janus Laust Thomsen, Tamana Afzali, Martin Bach Jensen

**Affiliations:** 1 Center for General Practice at Aalborg University, Fyrkildevej 7, 1. Sal, 9220 Aalborg Ø, Denmark; 2 Department of Sports Science and Clinical Biomechanics, Center for Muscle and Joint Health, University of Southern Denmark, Campusvej 55, 5230 Odense M, Denmark; 3 Nordic Institute of Chiropractic and Clinical Biomechanics, Campusvej 55, 5230 Odense, Denmark

**Keywords:** Low back pain, Feasibility study, General practice, Recruitment, Retention, Medical informatics app, Evidence-based-treatment

## Abstract

**Objective:**

In a future full-scale randomised controlled trial, we plan to compare satisfaction with a standard website versus satisfaction with a participatory driven web-application. The participatory driven web-application may facilitate the delivery of targeted evidence-based advice and information to patients with low back pain in general practice (ClinicalTrials.gov Identifier: NCT03088774). This feasibility study is intended to inform a future randomised controlled trial. The aim is to report on the lessons learned from recruitment to report on reasons for loss to follow-up.

**Results:**

We recruited 12 women and 8 men from two general practices with each practice recruiting for 3 months. Full follow-up data was available in only three patients (15%). Based on the high loss to follow-up, we do not consider it feasible to conduct the full-scale confirmatory trial as planned. Modifying inclusion criteria to include only patients expressing an interest in using online health information or randomising patients directly at the general practice, supporting them in accessing the web-application, and letting patients respond with their immediate satisfaction may improve the speed of recruitment and follow-up rates. Furthermore, the participatory driven web-application can be included in a larger multi-faceted intervention, making the combined intervention seem more relevant to study participants.

## Introduction

Low Back Pain (LBP) is a common symptom across cultures and affects people of all ages. It is the largest contributor to years lived with disability globally, and countries struggle to meet demands for healthcare [1]. General practice is often the first point of contact for patients with LBP [2], where addressing biological, psychological, and social aspects in the first line management is essential to reduce the impact of LBP on the individual [3]. Guidelines consistently recommend all patients to receive information about the nature of their pain and support to stay active and at work [4, 5]. Delivery of such information can be time consuming and therefore challenging for general practitioners (GPs) [6]. Consequently, information and advice according to guidelines are often not delivered, and implementation research to address this evidence-practice gap in general practice is needed [7]. We published the protocol for a large-scale randomised controlled trial, where we aim to compare a standard website versus a participatory driven web-application for patients with LBP to facilitate delivery of evidence-based advice and information [8].

The website was developed together with GPs, students and researchers [8]. First a number of student projects (24 in total) at Health Informatics at Aalborg University, Denmark, generated initial suggestions for designing the web application. Then, based on a synthesis of the suggestions, a semi-structured interview guide was made, which we used as the basis for 15 single-persons interviews of patients who had previously consulting a GP for LBP [9]. Following this, a workshop was conducted with seven patients with LBP, of which two had participated in the interview study. An early version of the new website was then presented and discussed with eight GPs during interviews to ensure that the website was feasible in routine management of LBP [10]. Finally, we showed the early version of the website to 150 patients with LBP [11] and inquired about readability, customisation, design, credibility, and usability [11]. The new participant-driven web-application contain guideline concordant information, support to stay active, patient stories, and exercise examples. The material is presented as text, pictures, and a short video of a GP advising about LBP. In a future randomised controlled trial we will compare patients’ satisfaction with this new participant-driven web-application with a standard reference website also containing guideline-based information on LBP [8].

This feasibility study is intended to inform the future randomised control trial [8]. The aim is thus to report on the lessons learned from the feasibility study with a focus on recruitment success and reasons for loss to follow-up.

## Main text

### Methods

We recruited 20 patients ≥ 18 years old who consulted two Danish general practices with acute or chronic LBP with or without concomitant leg pain [8]. Patients with spinal stenosis or serious underlying disease (e.g. signs of fracture, cauda equina syndrome, malignancy, osteoporosis, or spondyloarthritis), patients without Danish reading skills, pregnant women, and patients without access to the internet were not included [8]. However, it was not required that patients had an interest in receiving information online.

The GP invited the patient to participate. Patients were told that the purpose was to test the setup of the study, including the randomisation module and data collection. Patients were informed that the research team needed their contact information (phone number and e-mail address) to discuss any challenges with access to the website or with using online information to read about their LBP and finally to fill out an online questionnaire [8]. Patients were informed that the researchers would not report on their health-related clinical outcomes, e.g. reduction in pain or improved functional ability.

The Danish NemID was required to login from home [12]. NemID is a secure login system to the Internet and is typically applied for online banking and engagement with public authorities. When applying NemID three things are required: a user ID, a password, and a code card (either stored on a smartphone or in paper format in the size of a credit card) [12]. At the first login at home, the patient was again given information about the study. Consenting patients were then randomly allocated to the new website or to the online ‘Patient Handbook’ (standard website) [13]. Allocation was conducted online in blocks of two, four, and six, to the new web application or to the online ‘Patient Handbook’ [8]. The allocation sequence was delivered by a statistician at Aalborg University Hospital and integrated into the overall web application of the project [8]. After allocation, participants were asked to complete a longer online questionnaire at baseline, shorter online questionnaires during 5 days, and one longer online questionnaire at the end of the feasibility study (after 7 days) as well as a short questionnaire every day for 1 week. The shorter questionnaires contained 10 questions about satisfaction with the online information. We used the safe data storage and collection instrument, RedCap [14], to collect questionnaire data. Participants not accessing the website were sent reminders, and participants who dropped out of the study were phoned by the primary investigator (AR), who collected information about the reasons why.

## Results

**Fig. 1:**
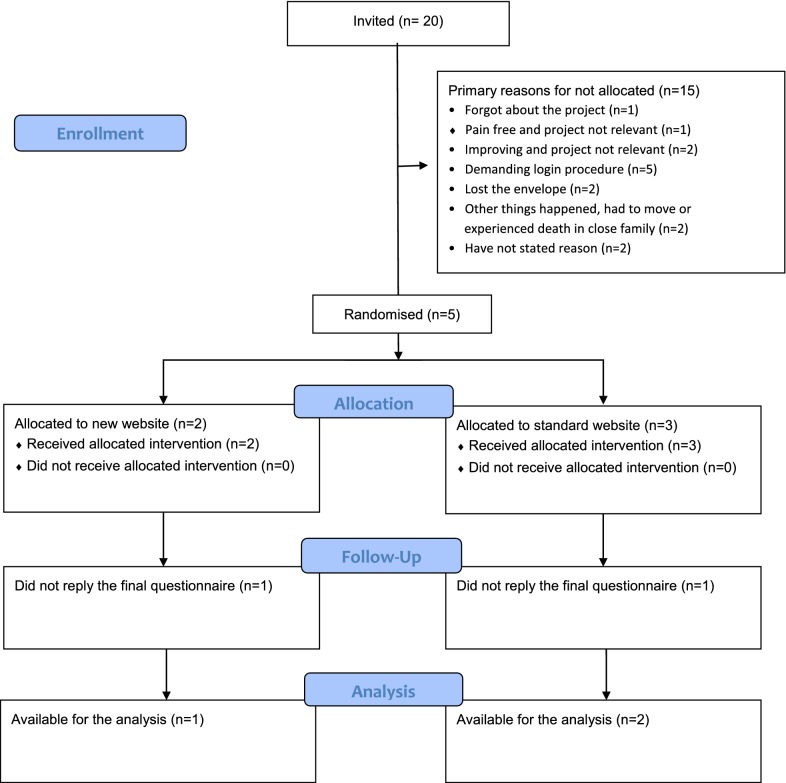
Flow chart

From December 18 2018 to May 1 2019, we recruited 20 patients from two general practices with each clinic recruiting for 3 months. Nine patients reported to have opened the project envelope delivered by their GP, but only five patients logged on to the project database and was allocated (two to the new web application and three to the ‘Patient Handbook’). Among the five allocated patients three completed the final questionnaire (one in the new application group and two in the ‘Patient Handbook’ group) (Fig. 1).

After all participants had been included for 1 week, they were contacted by phone to discuss reasons for dropping out.

### Lessons learned

Based on the high loss of participants, we do not believe it is feasible to conduct the full-scale randomised trial using the current protocol. Consequently, a redesign is needed followed by further testing of procedures and of the website. One approach could be to test the immediate effects among patients with a special interest in using a website as part of the management of their LBP. We did test an early version of the new website on 150 patients with the primary purpose to validate a questionnaire [11], and included patients familiar with using the Internet or the use of other web or applications to manage their pain. In that test, recruitment of 150 participants was not difficult highlighting that using the internet as a source of information may not be attractive for everyone. In a future randomised controlled trial, such a population could probably be recruited. In addition, the immediate satisfaction with the website could be assessed by having a healthcare provider or researcher present while patients access the information material, and immediate response on their satisfaction could be obtained. Giving the healthcare provider or researcher the opportunity to support patients in responding to questionnaires.

Another solution could be to evaluate the website as part of a larger multi-faceted intervention involving clinical staff members (other than GPs) in the management of LBP in general practice [15]. The rationale for including clinical staff is that they can assist in delivering key treatment elements to patients with LBP, like information about their pain condition and support of their self-management [15]. Having these issues thoroughly discussed with a clinical staff member may facilitate patients to be aware of the importance of these issues, facilitate participants’ interest in their study, and reduce drop-out.

## Limitations

In this feasibility study, we only included 20 patients, and we are therefore not able to draw conclusions about the potential effect of the intervention. This project used the Danish NEMid login, which is a common method to secure safe delivery of information between citizens in Denmark and public organizations. When patients receive a mail requiring NEMid, they expect this to be important information, but access is also a little cumbersome. Avoiding the use of NEMid might have improved follow-up, however, hardly to our required minimum level of 61% [8]. Hence, conducting the study with a more user-friendly access may speed up recruitment and support retention to the intervention.

## Data Availability

The dataset analysed during the current study will be available from the corresponding author on reasonable request. After study completion study completion, data will be kept will full availability for AR and MBJ. After 5 years, all data will be fully anonymised and kept at the Center for General Practice at Aalborg University.

## Abbreviations

GP: general practitioner
LBP: low back pain
